# Social Perception of Obesity – A 2025 Nationwide Cross-Sectional Survey Among Adults in Poland

**DOI:** 10.3389/ijph.2026.1608911

**Published:** 2026-02-19

**Authors:** Kuba Bartłomiej Sękowski, Agnieszka Mazurek, Zuzanna Grześczyk-Nojszewska, Mateusz Jankowski, Agnieszka Kamińska, Agata Olearczyk, Andrzej Silczuk, Justyna Grudziąż-Sękowska

**Affiliations:** 1 School of Public Health, Centre of Postgraduate Medical Education, Warsaw, Poland; 2 Faculty of Medicine, Collegium Medicum, Cardinal Stefan Wyszynski University, Warsaw, Poland; 3 Health Innovation Unit, SGH Warsaw School of Economics, Warsaw, Poland; 4 Department of Community Psychiatry, Medical University of Warsaw, Warsaw, Poland

**Keywords:** obesity, Poland, public beliefs, social perception, stigmatization

## Abstract

**Objectives:**

Obesity is a chronic, multifactorial disease. This study aimed to assess public attitudes towards obesity and identify factors influencing its social perception in a representative adult sample in Poland.

**Methods:**

A cross-sectional questionnaire-based survey of 1,088 Polish adults was conducted from 23–26 May 2025, using computer-assisted web interviewing (CAWI).

**Results:**

Most respondents (85.7%) considered obesity a significant health problem in Poland. Nearly half (45.5%) believed obese individuals showed less interest in their health, while 44.2% linked obesity to a lack of health concern. Additionally, 43.2% viewed obesity as a cause for shame. Support for greater societal acceptance of obesity was declared by 45.6%. Multivariable analysis showed that having children increased the odds of recognizing obesity as a major health issue (aOR = 1.58; 95%CI:1.06–2.36, p = 0.03). Men and younger adults were more likely to perceive obese people as less health-conscious (p < 0.05). Viewing obesity as shameful was associated with male gender (aOR = 1.48, 95%CI:1.16–1.91, p = 0.002), age 30–49, higher education (aOR = 1.44, 95%CI:1.11–1.87, p = 0.006), and living in towns of 20,000–499,999 residents.

**Conclusion:**

Obesity is perceived as a significant health problem, but mis-perceptions remain common.

## Introduction

According to the International Classification of Diseases (11th revision), obesity is a chronic disease of multifactorial etiology characterized by excess body fat that affects the body’s functioning [1].

In 2025, 16% of people globally (892 million) are predicted to be obese, and the numbers are estimated to reach up to 18% of the world’s population, which is over 1 billion people, by 2030 [2]. In addition, there is growing concern for the rapid increase in child and adolescent obesity, which has reached 8.5% globally and continues to rise [3].

While direct costs of overweight and obesity have already reached 2.19% of global GDP [4], the indirect costs are far higher, and the trend is experienced globally. The largest indirect costs contribute to premature mortality, absenteeism, and presenteeism [5, 6]. It has a wide impact on the country’s economy and society, resulting in productivity loss [7], lower earnings of individuals with higher healthcare costs, and higher social transfer payments [8]. This in turn generates further consequences in many areas of patients’ lives, both economic, social, and personal. The mental burden due to experienced stigma from coworkers, society, and even healthcare professionals results in lower spending, thus decreasing the quality of life in general [9, 10].

The occurrence and progression of obesity are partly dependent on modifiable lifestyle factors such as nutrition, physical activity, and smoking; however, the causes of obesity are much more numerous and not fully understood [11–13].

As a result of the association of obesity with lifestyle and questioning its status as a disease, in recent years there has been an increase in prejudice and stigmatization of people with obesity [14, 15], which is manifested, among others, through the presentation of negative, judgmental content in the media and discrimination on the labor market, in the education process, and contacts with the health service [16].

In response to this, phenomena such as body shaming (negative opinions expressed about someone’s appearance, often taking the form of offensive comments about body shape and weight) [17, 18] and the body positivity movement, encouraging acceptance of one’s own body, have developed [19].

It has been proven that social perceptions of obesity have a significant impact on the mental and physical health of people with obesity. Feeling prejudiced due to weight increases the risk of depressive symptoms, increased cortisol levels, emotional eating, and weight gain [20], discourages physical activity, and results in worse treatment outcomes [21].

Obesity is one of the most serious public health problems, which is the subject of action by many institutions around the world [22].

However, it is also necessary to describe the social image of obesity, which will allow for understanding social attitudes and the appropriate adjustment of public policies to effectively reduce and treat obesity.

Prior research shows that public perceptions of obesity often include stigmatizing beliefs, strong attributions of personal responsibility, and ambivalent support for social acceptance, with substantial cross-country variation [14–21]. However, evidence from Central and Eastern Europe remains limited, and recent epidemiological data on the prevalence of overweight and obesity from Poland are alarming [2]. Therefore, our study provides updated nationwide estimates of key perceptions and identifies factors associated with stigmatizing and supportive attitudes in a representative sample of Polish adults.

This study aimed to characterize public attitudes towards obesity and identify factors associated with differences in social perceptions of obesity in a representative sample of adults in Poland.

## Methods

### Study Design

This study was a cross-sectional survey of the Polish adult population. The survey was conducted between 23 May and 26 May 2025, using the computer-assisted web interviewing (CAWI) methodology. The authors of this study developed the study questionnaire and contracted Nationwide Research Panel Ariadna [23], a panel management company, to assist in administering the survey. The principles of the Declaration of Helsinki were followed throughout the study. Participation was voluntary, and informed consent was collected from all participants at the beginning of the study. The survey responses were anonymous. The study protocol was approved by the Ethics Committee at the Centre of Postgraduate Medical Education (Warsaw, Poland; decision no. 41/2025, 14 May 2025).

### Study Sample

The study sample was selected from a pool of 100,000 panelists through quota sampling. Therefore, the resulting sample was representative of the general adult population of Poland in terms of gender, age, and place of residence [24]. These three variables are most commonly used in stratification models in public opinion surveys in Poland. Respondents who returned incomplete responses were excluded from the study and replaced with individuals from the same stratum, so the minimum sample size of 1000 respondents could be reached.

### Measures

The authors of this study wrote the questionnaire based on a literature review [25–28]. The questionnaire comprised ten questions, of which four were about the knowledge of obesity as a disease and six were about the social perception of obesity. Only the questions relating to social perception were analyzed in this publication.

All six questions were five-point Likert items. For five agreement questions, the Likert scale ranged from “definitely yes” to “definitely no.” For one frequency question, the scale ranged from “yes, very often” to “no.” The responses “definitely yes” and “rather yes,” as well as “yes, very often”, “yes, often”, and “yes, rarely,” were aggregated into a single “yes” response for each question to facilitate analysis.

Respondents were also required to answer a series of questions regarding their education level, marital status, household size, occupational status, and economic status to make analysis by sociodemographic characteristics possible. In addition, respondents were asked to provide their height (cm) and weight (kg). This data was used to calculate the Body Mass Index (BMI) and assign respondents to one of the four BMI categories defined as follows: underweight (BMI <18.5), healthy weight (BMI ≥18.5), overweight (BMI ≥25), and obese (BMI ≥30) [29].

A pilot study involving 15 adults from the general population (non-medical background) was conducted to assess the clarity and relevance of the questionnaire items. Participants completed the questionnaire twice, with a 7-day interval between administrations. Responses from both time points were compared to evaluate item content and wording, and to identify any stylistic issues. Following this process, one item was removed, two items were revised, and four response options in multiple-choice questions were modified.

### Data Analysis

The IBM SPSS Statistics software (v. 29, Armonk, NY, United States) was used for data analysis. The survey results were presented in tables using response counts and frequencies. The associations between sociodemographic characteristics of participants and their questionnaire responses were tested using the chi-square test of independence and logistic regression. Only those variables that showed statistical significance in bivariable logistic regression were included in the multivariable logistic regression models. Logistic regression results were reported using odds ratios (OR) and 95% confidence intervals (95% CI). Statistical significance was set at p < 0.05.

## Results

### Characteristics of the Study Population

In total, 1088 individuals were included in the study. Of the participants, 46% were male, and 54% were female. The age distribution was as follows: 13.3% were aged 18 to 29, 19.8% were aged 30 to 39, 19.4% were aged 40 to 49, 17.5% were aged 50 to 59, and 30.1% were aged 60 or older. 38.1% of participants lived in rural areas, and 61.9% lived in urban areas.

Out of the 1088 participants, the majority had less than higher education (53.5%), were occupationally active (64.1%), were married (53.0%), and had children (64.5%). Regarding household size, 15.7% of participants lived alone, 38.4% lived in a household of two, and 45.9% lived in a household of three or more. Nearly half of the participants reported a good economic status (49.6%), while 36.7% reported a moderate status, and 13.7% reported a bad status. Regarding BMI categorization, 2.8% of participants were classified as underweight, 37.9% as overweight, and 20.2% as obese, while 39.2% had a healthy weight.

### Social Perception of Obesity

Overall, 85.7% of participants believed that obesity is a significant health problem in Poland, with 41.6% answering “definitely yes” and 44.1% answering “rather yes” (Table 1). However, a substantial proportion of participants, specifically 14.9% (“definitely yes”) and 30.7% (“rather yes”), thought that overweight and obesity should be more widely accepted in society as a natural body type rather than a health problem (Table 1). In response to whether obese people show less interest in their health than people with a healthy weight, only 13.8% of participants answered “definitely yes”, while 31.7% answered “rather yes” (Table 1). Less than half of the participants endorsed (10.8% “definitely yes” and 33.4% “rather yes”) the statement that obesity results from a lack of interest in one’s health (Table 1). Similarly, 12.1% (“definitely yes”) and 31.1% (“rather yes”) of participants indicated that obesity is a cause for shame (Table 1). Only 7.7% of participants indicated that they very often encounter situations in which an obese person is treated with disrespect in a public space. Meanwhile, 16.8% indicated that they witness such occurrences often, and 23.8% indicated they encounter them, albeit rarely (Table 1).

**TABLE 1:** Social perception of obesity in the opinion of adult residents of Poland (N = 1088) (Warsaw, Poland, 2025).

Variable	n	%
In your opinion, is obesity a significant health problem in Poland?		
Definitely yes	453	41.6
Rather yes	480	44.1
Rather no	61	5.6
Definitely no	6	0.6
I do not know/difficult to tell	88	8.1
Do you think that obese people show less interest in their health than people with a healthy weight?		
Definitely yes	150	13.8
Rather yes	345	31.7
Rather no	231	21.2
Definitely no	91	8.4
I Do not know/difficult to tell	271	24.9
Do you agree with the statement that obesity results from a lack of interest in one’s health?		
Definitely yes	118	10.8
Rather yes	363	33.4
Rather no	278	25.6
Definitely no	91	8.4
I do not know/difficult to tell	238	21.9
Do you think obesity is a cause for shame?		
Definitely yes	132	12.1
Rather yes	338	31.1
Rather no	287	26.4
Definitely no	137	12.6
I do not know/difficult to tell	194	17.8
Do you think that overweight and obesity should be more widely accepted in society as a natural body type rather than a health problem?		
Definitely yes	162	14.9
Rather yes	334	30.7
Rather no	234	21.5
Definitely no	128	11.8
I do not know/difficult to tell	230	21.1
Have you encountered a situation in which an obese person was treated with disrespect in a public space (e.g., in a store or on public transport)?		
Yes, very often	84	7.7
Yes, often	183	16.8
Yes, rarely	259	23.8
No	333	30.6
I Do not know/difficult to tell	229	21.0

### Sociodemographic Differences in Social Perception of Obesity

There were statistically significant differences in the social perception of obesity by sociodemographic variables (Tables 2–6).

**TABLE 2:** Factors associated with perception of obesity as a significant health problem in Poland (N = 1088) (Warsaw, Poland, 2025).

In your opinion, is obesity a significant health problem in Poland? – „Rather yes” or „definitely yes”						
Variable	%	p	Bivariable logistic Regression		Multivariable logistic Regression	
			OR (95%CI)	p	aOR (95%CI)	p
Gender						
Female (n = 588)	86.7	0.3	1.19 (0.85–1.67)	0.3	​	​
Male (n = 500)	84.6	​	Reference	​	​	​
Age group [years]						
18–29 (n = 145)	85.5	0.2	0.82 (0.47–1.45)	0.5	1.41 (0.72–2.75)	0.3
30–39 (n = 215)	84.2	​	0.74 (0.45–1.22)	0.2	1.01 (0.59–1.73)	0.9
40–49 (n = 211)	81.5	​	0.62 (0.38–0.99)	0.04	0.77 (0.46–1.27)	0.3
50–59 (n = 190)	88.9	​	1.12 (0.64–1.97)	0.7	1.20 (0.68–2.12)	0.5
60+ (n = 327)	87.8	​	Reference	​	Reference	​
Educational level						
Higher (n = 506)	87.9	0.05	1.41 (0.99–1.99)	0.06	​	​
Less than higher (n = 582)	83.8	​	Reference	​	​	​
Married						
Yes (n = 577)	87.5	0.08	1.36 (0.97–1.91)	0.08	​	​
No (n = 511)	83.8	​	Reference	​	​	​
Place of residence						
Rural area (n = 415)	85.5	0.4	1.28 (0.76–2.15)	0.4	​	​
City below 20,000 residents (n = 144)	83.3	​	1.08 (0.58–2.01)	0.8	​	​
City from 20,000 to 99,999 residents (n = 212)	88.2	​	1.62 (0.88–2.97)	0.1	​	​
City from 100,000 to 499,999 residents (n = 182)	87.9	​	1.57 (0.84–2.94)	0.2	​	​
City ≥500,000 residents (n = 135)	82.2	​	Reference	​	​	​
Having children						
Yes (n = 702)	87.7	0.01	1.56 (1.11–2.20)	0.01	1.58 (1.06–2.36)	0.03
No (n = 386)	82.1	​	Reference	​	Reference	​
Number of household members						
1 (living alone) (n = 171)	81.9	0.2	0.77 (0.49–1.23)	0.3	​	​
2 (n = 418)	87.8	​	1.23 (0.84–1.81)	0.3	​	​
3 or more (n = 499)	85.4	​	Reference	​	​	​
Occupational status						
Active (n = 668)	85.5	0.7	0.94 (0.66–1.34)	0.7	​	​
Passive (n = 420)	86.2	​	Reference	​	​	​
Self-reported household economic status						
Good (n = 540)	89.3	0.004	1.60 (0.95–2.67)	0.08	​	​
Moderate (n = 399)	81.7	​	0.86 (0.52–1.42)	0.6	​	​
Bad (n = 149)	83.9	​	Reference	​	​	​
BMI group						
Underweight (n = 30)	70.0	0.02	0.45 (0.20–1.03)	0.06	0.45 (0.19–1.04)	0.06
Overweight (n = 412)	86.9	​	1.28 (0.87–1.88)	0.2	1.21 (0.81–1.79)	0.4
Obesity (n = 220)	89.5	​	1.65 (1.01–2.74)	0.04	1.59 (0.95–2.66)	0.08
Healthy weight (n = 426)	83.8	​	Reference	​	Reference	​

**TABLE 3:** Factors associated with perception of obese people as less interested in their health than people with a healthy weight (N = 1088) (Warsaw, Poland, 2025).

Do you think that obese people show less interest in their health than people with a healthy weight? – „Rather yes” or „definitely yes”						
Variable	%	p	Bivariable logistic Regression		Multivariable logistic Regression	
			OR (95%CI)	p	aOR (95%CI)	p
Gender						
Female (n = 588)	41.2	0.002	Reference	0.002	Reference	0.002
Male (n = 500)	50.6	​	1.46 (1.15–1.86)	​	1.50 (1.16–1.93)	​
Age group [years]						
18–29 (n = 145)	46.2	<0.001	1.70 (1.14–2.53)	0.01	1.68 (1.07–2.65)	0.03
30–39 (n = 215)	58.1	​	2.74 (1.92–3.91)	<0.001	2.43 (1.59–3.70)	<0.001
40–49 (n = 211)	46.0	​	1.68 (1.18–2.39)	0.004	1.48 (0.96–2.27)	0.07
50–59 (n = 190)	50.5	​	2.02 (1.40–2.90)	<0.001	2.00 (1.32–3.02)	0.001
60+ (n = 327)	33.6	​	Reference	​	Reference	​
Educational level						
Higher (n = 506)	48.8	0.04	1.28 (1.01–1.63)	0.04	1.22 (0.95–1.57)	0.1
Less than higher (n = 582)	42.6	​	Reference	​	Reference	​
Married						
Yes (n = 577)	46.4	0.5	1.09 (0.85–1.38)	0.5	​	​
No (n = 511)	44.4	​	Reference	​	​	​
Place of residence						
Rural area (n = 415)	45.1	0.5	1.23 (0.83–1.83)	0.3	​	​
City below 20,000 residents (n = 144)	43.8	​	1.17 (0.73–1.88)	0.5	​	​
City from 20,000 to 99,999 residents (n = 212)	49.5	​	1.47 (0.95–2.28)	0.08	​	​
City from 100,000 to 499,999 residents (n = 182)	47.3	​	1.34 (0.86–2.11)	0.2	​	​
City ≥500,000 residents (n = 135)	40.0	​	Reference	​	​	​
Having children						
Yes (n = 702)	45.0	0.7	0.95 (0.74–1.22)	0.7	​	​
No (n = 386)	46.4	​	Reference	​	​	​
Number of household members						
1 (living alone) (n = 171)	41.5	0.07	0.73 (0.51–1.04)	0.08	0.89 (0.61–1.30)	0.5
2 (n = 418)	42.6	​	0.76 (0.59–0.99)	0.04	0.96 (0.72–1.29)	0.8
3 or more (n = 499)	49.3	​	Reference	​	Reference	​
Occupational status						
Active (n = 668)	50.4	<0.001	1.69 (1.32–2.17)	<0.001	1.10 (0.81–1.51)	0.5
Passive (n = 420)	37.6	​	Reference	​	Reference	​
Self-reported household economic status						
Good (n = 540)	48.0	0.2	1.07 (0.74–1.54)	0.7	​	​
Moderate (n = 399)	41.9	​	0.84 (0.57–1.22)	0.3	​	​
Bad (n = 149)	46.3	​	Reference	​	​	​
BMI group						
Underweight (n = 30)	36.7	0.06	0.64 (0.30–1.37)	0.2	0.68 (0.31–1.50)	0.3
Overweight (n = 412)	47.8	​	1.01 (0.77–1.32)	0.9	1.06 (0.79–1.42)	0.7
Obesity (n = 220)	38.2	​	0.68 (0.49–0.95)	0.02	0.72 (0.50–1.02)	0.07
Healthy weight (n = 426)	47.7	​	Reference	​	Reference	​

**TABLE 4:** Factors associated with agreement with the statement that obesity results from a lack of interest in one’s health (N = 1088) (Warsaw, Poland, 2025).

Do you agree with the statement that obesity results from a lack of interest in one’s health? – „Rather yes” or „definitely yes”						
Variable	%	p	Bivariable logistic Regression		Multivariable logistic Regression	
			OR (95%CI)	p	aOR (95%CI)	p
Gender						
Female (n = 588)	37.8	<0.001	Reference	<0.001	Reference	<0.001
Male (n = 500)	51.8	​	1.77 (1.39–2.26)	​	1.94 (1.51–2.50)	​
Age group [years]						
18–29 (n = 145)	52.4	0.2	1.57 (1.06–2.32)	0.03	1.53 (1.02–2.32)	0.04
30–39 (n = 215)	46.5	​	1.24 (0.87–1.75)	0.2	1.13 (0.79–1.62)	0.5
40–49 (n = 211)	43.1	​	1.08 (0.76–1.53)	0.7	0.97 (0.67–1.39)	0.9
50–59 (n = 190)	41.6	​	1.01 (0.70–1.46)	0.9	1.06 (0.73–1.53)	0.8
60+ (n = 327)	41.3	​	Reference	​	Reference	​
Educational level						
Higher (n = 506)	42.7	0.4	0.89 (0.70–1.13)	0.3	​	​
Less than higher (n = 582)	45.5	​	Reference	​	​	​
Married						
Yes (n = 577)	43.7	0.7	0.96 (0.75–1.21)	0.7	​	​
No (n = 511)	44.8	​	Reference	​	​	​
Place of residence						
Rural area (n = 415)	46.3	0.5	1.11 (0.75–1.64)	0.6	​	​
City below 20,000 residents (n = 144)	41.0	​	0.89 (0.56–1.44)	0.6	​	​
City from 20,000 to 99,999 residents (n = 212)	46.7	​	1.13 (0.73–1.74)	0.6	​	​
City from 100,000 to 499,999 residents (n = 182)	39.6	​	0.84 (0.54–1.32)	0.5	​	​
City ≥500,000 residents (n = 135)	43.7	​	Reference	​	​	​
Having children						
Yes (n = 702)	42.5	0.1	0.82 (0.64–1.05)	0.1	​	​
No (n = 386)	47.4	​	Reference	​	​	​
Number of household members						
1 (living alone) (n = 171)	45.6	0.6	1.01 (0.72–1.44)	0.9	​	​
2 (n = 418)	42.3	​	0.89 (0.68–1.15)	0.4	​	​
3 or more (n = 499)	45.3	​	Reference	​	​	​
Occupational status						
Active (n = 668)	45.8	0.2	1.18 (0.93–1.51)	0.2	​	​
Passive (n = 420)	41.7	​	Reference	​	​	​
Self-reported household economic status						
Good (n = 540)	50.0	<0.001	1.29 (0.90–1.86)	0.2	​	​
Moderate (n = 399)	36.6	​	0.75 (0.51–1.09)	0.1	​	​
Bad (n = 149)	43.6	​	Reference	​	​	​
BMI group						
Underweight (n = 30)	43.3	0.01	0.86 (0.41–1.81)	0.7	0.88 (0.41–1.89)	0.7
Overweight (n = 412)	46.4	​	0.97 (0.74–1.27)	0.8	0.90 (0.68–1.20)	0.5
Obesity (n = 220)	34.5	​	0.59 (0.42–0.83)	0.002	0.54 (0.38–0.77)	<0.001
Healthy weight (n = 426)	47.2	​	Reference	​	Reference	​

**TABLE 5:** Factors associated with perception of obesity as a cause for shame (N = 1088) (Warsaw, Poland, 2025).

Do you think obesity is a cause for shame? – „Rather yes” or „definitely yes”						
Variable	%	p	Bivariable logistic Regression		Multivariable logistic Regression	
			OR (95%CI)	p	aOR (95%CI)	p
Gender						
Female (n = 588)	38.6	<0.001	Reference	<0.001	Reference	0.002
Male (n = 500)	48.6	​	1.50 (1.18–1.91)	​	1.48 (1.16–1.91)	​
Age group [years]						
18–29 (n = 145)	42.1	0.007	1.29 (0.86–1.92)	0.2	1.42 (0.90–2.22)	0.1
30–39 (n = 215)	50.7	​	1.82 (1.28–2.58)	<0.001	1.81 (1.18–2.76)	0.006
40–49 (n = 211)	48.3	​	1.66 (1.17–2.36)	0.005	1.68 (1.09–2.59)	0.02
50–59 (n = 190)	42.1	​	1.29 (0.89–1.86)	0.2	1.37 (0.90–2.09)	0.1
60+ (n = 327)	36.1	​	Reference	​	Reference	​
Educational level						
Higher (n = 506)	48.0	0.003	1.45 (1.14–1.84)	0.003	1.44 (1.11–1.87)	0.006
Less than higher (n = 582)	39.0	​	Reference	​	Reference	​
Married						
Yes (n = 577)	44.9	0.2	1.16 (0.91–1.47)	0.2	​	​
No (n = 511)	41.3	​	Reference	​	​	​
Place of residence						
Rural area (n = 415)	42.7	0.2	1.26 (0.85–1.89)	0.3	1.47 (0.96–2.26)	0.08
City below 20,000 residents (n = 144)	39.6	​	1.11 (0.69–1.81)	0.7	1.26 (0.76–2.08)	0.4
City from 20,000 to 99,999 residents (n = 212)	45.8	​	1.43 (0.92–2.23)	0.1	1.59 (1.01–2.52)	0.04
City from 100,000 to 499,999 residents (n = 182)	48.9	​	1.63 (1.03–2.56)	0.04	1.96 (1.22–3.14)	0.005
City ≥500,000 residents (n = 135)	37.0	​	Reference	​	Reference	​
Having children						
Yes (n = 702)	43.0	0.9	0.98 (0.76–1.26)	0.9	​	​
No (n = 386)	43.5	​	Reference	​	​	​
Number of household members						
1 (living alone) (n = 171)	36.3	0.1	0.69 (0.48–0.99)	0.04	0.81 (0.55–1.20)	0.3
2 (n = 418)	43.8	​	0.95 (0.73–1.23)	0.7	1.16 (0.86–1.55)	0.3
3 or more (n = 499)	45.1	​	Reference	​	Reference	​
Occupational status						
Active (n = 668)	46.4	0.007	1.41 (1.10–1.81)	0.007	1.00 (0.73–1.38)	0.9
Passive (n = 420)	38.1	​	Reference	​	Reference	​
Self-reported household economic status						
Good (n = 540)	46.3	0.01	0.95 (0.66–1.36)	0.8	0.90 (0.61–1.33)	0.6
Moderate (n = 399)	37.3	​	0.66 (0.49–0.96)	0.03	0.64 (0.43–0.96)	0.03
Bad (n = 149)	47.7	​	Reference	​	Reference	​
BMI group						
Underweight (n = 30)	30.0	0.3	0.54 (0.24–1.20)	0.1	​	​
Overweight (n = 412)	41.3	​	0.88 (0.67–1.16)	0.4	​	​
Obesity (n = 220)	46.4	​	1.08 (0.78–1.50)	0.6	​	​
Healthy weight (n = 426)	44.4	​	Reference	​	​	​

**TABLE 6:** Factors associated with opinion that overweight and obesity should be more widely accepted in society as a natural body type rather than a health problem or experience of disrespect of obese people in a public space (N = 1088) (Warsaw, Poland, 2025).

Do you think that overweight and obesity should be more widely accepted in society as a natural body type rather than a health problem? – „rather yes” or „definitely yes”							Have you encountered a situation in which an obese person was treated with disrespect in a public space? – “yes, very often”, „yes, often”, „yes, rarely”					
Variable	%	p	Bivariable Logistic Regression		Multivariable Logistic Regression		%	p	Bivariable Logistic Regression		Multivariable Logistic Regression	
			OR (95%CI)	p	aOR (95%CI)	p			OR (95%CI)	p	aOR (95%CI)	p
Gender												
Female (n = 588)	48.0	0.09	1.23 (0.97–1.57)	0.09	​	​	45.4	0.9	0.98 (0.78–1.25)	0.9	​	​
Male (n = 500)	42.8	​	Reference	​	​	​	45.8	​	Reference	​	​	​
Age group [years]												
18–29 (n = 145)	33.1	0.02	Reference	​	Reference	​	68.3	<0.001	4.55 (2.99–6.92)	<0.001	6.80 (3.98–11.60)	<0.001
30–39 (n = 215)	47.4	​	1.82 (1.18–2.83)	0.007	1.73 (1.08–2.75)	0.02	53.0	​	2.39 (1.67–3.40)	<0.001	2.75 (1.76–4.29)	<0.001
40–49 (n = 211)	45.0	​	1.66 (1.07–2.57)	0.03	1.39 (0.86–2.25)	0.2	41.7	​	1.51 (1.06–2.17)	0.02	1.68 (1.09–2.61)	0.02
50–59 (n = 190)	48.9	​	1.94 (1.24–3.03)	0.004	1.47 (0.89–2.43)	0.1	47.4	​	1.90 (1.32–2.75)	<0.001	1.87 (1.23–2.84)	0.003
60+ (n = 327)	48.3	​	1.89 (1.26–2.84)	0.002	1.45 (0.89–2.34)	0.1	32.1	​	Reference	​	Reference	​
Educational level												
Higher (n = 506)	39.1	<0.001	Reference	<0.001	Reference	<0.001	42.7	0.07	0.80 (0.63–1.02)	​	​	​
Less than higher (n = 582)	51.2	​	1.63 (1.28–2.08)	​	1.54 (1.19–1.99)	​	48.1	​	Reference	​	​	​
Married												
Yes (n = 577)	47.3	0.2	1.16 (0.91–1.47)	0.2	​	​	42.3	0.02	Reference	0.02	Reference	0.6
No (n = 511)	43.6	​	Reference	​	​	​	49.3	​	1.33 (1.05–1.69)	​	0.92 (0.68–1.24)	​
Place of residence												
Rural area (n = 415)	48.0	0.03	1.78 (1.19–2.67)	0.005	1.49 (0.98–2.27)	0.07	43.4	0.7	0.96 (0.65–1.42)	0.8	​	​
City below 20,000 residents (n = 144)	52.1	​	2.10 (1.30–3.41)	0.003	1.93 (1.17–3.18)	0.01	45.8	​	1.06 (0.66–1.70)	0.8	​	​
City from 20,000 to 99,999 residents (n = 212)	45.8	​	1.63 (1.04–2.56)	0.03	1.53 (0.97–2.43)	0.07	49.5	​	1.23 (0.80–1.89)	0.4	​	​
City from 100,000 to 499,999 residents (n = 182)	43.4	​	1.48 (0.94–2.35)	0.09	1.33 (0.82–2.13)	0.2	46.7	​	1.10 (0.70–1.71)	0.7	​	​
City ≥500,000 residents (n = 135)	34.1	​	Reference	​	Reference	​	44.4	​	Reference	​	​	​
Having children												
Yes (n = 702)	48.6	0.008	1.41 (1.10–1.81)	0.008	1.30 (0.96–1.76)	0.09	41.2	<0.001	Reference	<0.001	Reference	0.9
No (n = 386)	40.2	​	Reference	​	Reference	​	53.6	​	1.65 (1.29–2.12)	​	0.98 (0.70–1.37)	​
Number of household members												
1 (living alone) (n = 171)	45.0	0.7	0.93 (0.65–1.32)	0.7	​	​	40.9	0.4	0.79 (0.55–1.12)	0.2	​	​
2 (n = 418)	44.3	​	0.90 (0.69–1.17)	0.4	​	​	45.9	​	0.96 (0.74–1.25)	0.8	​	​
3 or more (n = 499)	46.9	​	Reference	​	​	​	46.9	​	Reference	​	​	​
Occupational status												
Active (n = 668)	43.6	0.09	0.81 (0.63–1.03)	0.09	​	​	48.4	0.02	1.34 (1.05–1.71)	0.02	0.97 (0.70–1.34)	0.9
Passive (n = 420)	48.8	​	Reference	​	​	​	41.2	​	Reference	​	Reference	​
Self-reported household economic status												
Good (n = 540)	40.2	<0.001	Reference	​	Reference	​	39.6	<0.001	Reference	​	Reference	​
Moderate (n = 399)	48.4	​	1.40 (1.07–1.81)	0.01	1.25 (0.95–1.64)	0.1	49.9	​	1.52 (1.17–1.97)	0.002	1.59 (1.20–2.10)	0.001
Bad (n = 149)	57.7	​	2.03 (1.41–2.94)	<0.001	1.86 (1.27–2.74)	0.002	55.7	​	1.92 (1.33–2.76)	<0.001	2.05 (1.38–3.04)	<0.001
BMI group												
Underweight (n = 30)	36.7	0.02	0.82 (0.38–1.77)	0.6	0.86 (0.39–1.90)	0.7	30.0	<0.001	0.52 (0.23–1.16)	0.1	0.31 (0.13–0.73)	0.007
Overweight (n = 412)	46.4	​	1.23 (0.93–1.61)	0.1	1.13 (0.85–1.50)	0.4	41.0	​	0.84 (0.64–1.10)	0.2	1.01 (0.75–1.36)	0.9
Obesity (n = 220)	53.6	​	1.64 (1.18–2.28)	0.003	1.44 (1.02–2.03)	0.04	56.8	​	1.59 (1.14–2.21)	0.006	1.97 (1.38–2.82)	<0.001
Healthy weight (n = 426)	41.3	​	Reference	​	Reference	​	45.3	​	Reference	​	Reference	​

There were sociodemographic differences in the perception of obesity as a significant health problem in Poland by having children, self-reported household economic status, and BMI category (Table 2). In multivariable logistic regression, having children was the only factor significantly associated with higher odds (aOR:1.58, 95%CI:1.06–2.36, p = 0.03) of indicating that obesity is a significant health problem in Poland (Table 2).

The perception of obese people as less interested in their health than those with a healthy weight differed according to gender, age, education level, and occupational status (Table 3). In multivariable logistic regression (Table 3), male gender was associated with higher odds (aOR:1.50, 95%CI:1.16–1.93, p = 0.002) of holding this perception. Furthermore, younger age groups had higher odds of perceiving obese individuals as less interested in their health compared with participants aged 60 and older. Specifically, participants aged 18–29 had an aOR of 1.68 (95%CI:1.07–2.65, p = 0.03), participants aged 30–39 had an aOR of 2.43 (95%CI:1.59–3.70, p < 0.001), and those aged 50–59 had an aOR of 2.00 (95%CI:1.32–3.02, p = 0.001).

The endorsement of the statement that obesity results from a lack of interest in one’s health varied by gender, self-reported economic status, and BMI category (Table 4). In multivariable logistic regression (Table 4), men had higher odds of agreeing with this statement (aOR:1.94, 95%CI:1.51–2.50, p < 0.001). Participants aged 18–29 also showed higher odds of agreement compared with those aged 60 and older (aOR:1.53, 95%CI: 1.02–2.32, p = 0.04). In contrast, the odds of agreeing that obesity stems from disinterest in one’s health were lower among participants with obesity compared with those with a healthy weight (aOR: 0.54, 95%CI:0.38–0.77, p < 0.001).

The perception of obesity as a cause for shame differed based on gender, age, education level, place of residence, and self-reported economic status (Table 5). In multivariable logistic regression (Table 5), higher odds of viewing obesity as shameful were observed for males aOR:1.48, 95%CI:1.16–1.91, p = 0.002), participants with higher education (aOR:1.44, 95%CI:1.11–1.87, p = 0.006), those aged 30–39 (aOR:1.81, 95%CI:1.18–2.76, p = 0.006) or 40–49 (aOR:1.68, 95%CI:1.09–2.59, p = 0.02), as well as participants living in cities with 20,000–99,999 residents (aOR:1.59, 95%CI:1.01–2.52, p = 0.04) or cities with 100,000–499,999 residents (aOR:1.96, 95%CI:1.22–3.14, p = 0.005). Conversely, having a moderate economic status, as opposed to bad status, was significantly associated with lower odds (aOR:0.64, 95%CI:0.43–0.96, p = 0.03) of perceiving obesity as a cause for shame.

There were sociodemographic differences in the opinion that overweight and obesity should be more widely accepted in society as a natural body type rather than a health problem by age, education level, place of residence, having children, self-reported economic status, and BMI category (Table 6). In multivariable logistic regression (Table 6), participants aged 30–39 had higher odds (aOR:1.73, 95%CI:1.08–2.75, p = 0.02) of expressing this opinion compared with those aged 18–29. The other factors significantly associated with higher odds of believing that overweight and obesity should be socially accepted as natural body types were having less than higher education (aOR:1.54, 95%CI:1.19–1.99, p < 0.001), living in cities with less than 20,000 residents (aOR:1.93, 95%CI:1.17–3.18, p = 0.01), and having a bad economic status as opposed to good status (aOR:1.86, 95%CI:1.27–2.74, p = 0.002). Higher odds of this belief were also observed among participants with obesity (aOR:1.44, 95%CI:1.02–2.03, p = 0.04).

The frequency of witnessing disrespect towards obese people in public spaces differed according to age, marital status, having children, occupational status, self-reported economic status, and BMI category (Table 6). In multivariable logistic regression (Table 6), participants in all four younger age groups, compared with those aged 60 and over, had higher odds of reporting such encounters, with participants aged 18–29 having the highest odds (aOR:6.80, 95%CI:3.98–11.60, p < 0.001). Similarly, there were higher odds of encountering disrespect towards obese people in public among participants with a moderate (aOR:1.59, 95%CI:1.20–2.10, p = 0.001) and bad (aOR:2.05, 95%CI:1.38–3.04, p < 0.001) economic status. Notably, obesity was significantly associated with higher odds (aOR:1.97, 95%CI:1.38–2.82, p < 0.001) of witnessing such situations, whereas being underweight was significantly associated with lower odds (aOR: 0.31, 95%CI:0.13–0.73, p = 0.007).

## Discussion

This is the most up-to-date study on social perception of obesity that was carried out in a representative sample of adults in Poland. The results showed the existence of incorrect attitudes and opinions in society about people with excess body weight. Obesity stigma is a phenomenon that is quite widespread in Poland. In multivariable logistics regression, males, those aged 30–49 years, individuals with higher education, and those living in cities with 20,000 to 499,999 residents were more likely (p < 0.05) to declare that obesity is a case for shame. Individuals aged 30–39 years, those without higher education, those living in cities below 20,000 residents as well as individuals with bad economic status were more likely (p < 0.05) to declare that overweight and obesity should be more widely accepted in society.

It is estimated that 56.6% of adults in Poland have excessive body weight (BMI >25 kg/m2) [30]. Worldwide, the prevalence of obesity is constantly increasing and causes a global public health challenge [31]. In this study, 85.7% of adults indicated that obesity is a significant health problem in Poland. As the number of people with excess body weight, including obesity, increases, the number of people who consider obesity to be a serious health problem may increase. Is finding. In multivariable logistic regression, only having children was a factor significantly associated with the beliefs that obesity is a significant health problem in Poland. This observation suggests that as excessive body mass in children is a growing problem, parents are more likely to notice the problem of obesity in society [32].

Findings from Singapore showed that 60.4% of the general public agreed that obesity was a disease and 80.5% believed that patients should be solely responsible for managing their obesity and weight [33]. Differences between our study and the study from Singapore [33] may result from some socio-cultural differences in the perception of obesity and the role of health systems in managing lifestyle-related diseases.

A study by Świder et al., performed among adults in Poland using social media platforms like Facebook.com and Instagram, showed that 74% of respondents believe that individuals with obesity are less attractive than those with normal body weight [34]. Moreover, 32.2% believed that obesity is a cause for shame [34]. Kindness (43.9%), willingness to help (34.0%), and compassion (33.4%) were the most common feelings accompanying interactions with people with obesity [34]. Moreover, Świder et al. noticed that patients with obesity in Poland may face discrimination [34]. A study by Baska et al., performed in 2023 among physicians, dietitians, and other healthcare professionals in Poland revealed a moderate level of fat phobia among Polish doctors and a comparatively lower level among other healthcare professionals [35]. However, Sobczak et al. showed that in 2020 in Poland, 82.6% of patients with obesity experienced inappropriate behaviors, often attributed to healthcare professionals [36].

Previously published studies on social perception of obesity in Poland [34–36], were carried out in non-representative samples of adults of healthcare professionals. This study is a nationwide cross-sectional study, with quota sampling methods and a stratification model (age, gender, place of residence), that produces representative data on social perception of obesity in a representative sample of adults in Poland. Findings from this study showed that almost half of adults in Poland believe that obese people show less interest in their health than people with a healthy weight (45.5%) and that obesity results from a lack of interest in one’s health (44.2%). Moreover, in this study, 43.2% of respondents declared that obesity is a cause of shame. This observation is in line with studies by Świder et al. [34] and Baska et al. [35], and points out the presence of obesity stigma in the Polish society.

Males and the younger respondents were more likely to hold stigmatizing views of obesity. Gender differences in misperception of body weight are well-documented [37, 38]. Moreover, those with higher education and individuals living in cities, from 20,000 to 499,999 residents were more likely to present stigmatizing views of obesity. These sociodemographic differences in social perception of obesity by gender, age, place of residence, and educational level should be addressed in educational campaigns and public health interventions, as they may aggravate health disparities and inequalities in Poland.

Adults aged 30–39 years, those without higher education, residents of small cities (<20,000 residents) as well as those with bad economic status were more likely to declare that overweight and obesity should be more widely accepted in society as a natural body type. This observation points out sociodemographic groups that are more likely to share the body positivity views [19].

In line with the guidelines published by the Polish Society for the Treatment of Obesity [39], stigmatization of excess body weight results in a deterioration in the wellbeing and mental health of obese patients, and thus translates into poorer treatment outcomes. In this study respondents under 60 years and those with moderate or poor economic status were more likely to witness disrespect towards obese people in public spaces. In general, 48.3% of adults in Poland witnessed this kind of disrespect. This observation underlines a serious public problem and suggests that psychological support should be offered to people who are under medical care due to obesity.

In this study, differences in social perception of obesity by BMI status were analyzed. Obese individuals were more likely than those with a healthy weight to support body-type acceptance and less likely to declare that obesity results from a lack of interest in one’s health. Moreover, compared with individuals with a healthy weight, obese individuals had more exposure to stigmatizing environments or were more sensitive to recognizing stigmatizing behaviors. However, in this study, there were no significant differences in social perception of obesity between those with a healthy weight and overweight. Findings from this study proved that people with obesity may be exposed to stigmatization and their needs are not fully addressed by public policies. Public health interventions on obesity should put more attention to the social aspects of the disease and provide psychological support that is crucial to maintain motivation to reduce body mass and change lifestyle.

Mata and Hertwig, in representative cross-sectional surveys conducted in the United States, the United Kingdom, and Germany, found that people attributed responsibility for obesity primarily to individual decisions (similar to those for alcohol and tobacco use) and personal responsibility [40]. In this study, most of the respondents also indicated that obesity is a result of individuals’ lifestyle. Findings from the WW International study conducted in six countries (Australia, Canada, France, Germany, the UK, and the US) by Puhl et al., showed that more than half of participants (55.6%–61.3%) across countries reported experiencing weight stigma, wherein participants with higher BMI were significantly more likely to report weight-stigmatizing experiences than individuals with lower BMI [41]. In this study, 48.3% of respondents declared that they had witnessed the stigmatization of people with obesity. In line with the study by Puhl et al. [41], those with obesity were more likely to declare past stigmatization experience due to body weight. A comparative study between Spain and Egypt (Sánchez et al.) demonstrated marked cross-cultural differences in obesity stigma [42]. Egyptian participants reported significantly higher aversion toward obesity [42]. Spanish participants reported more stigmatizing situations than Egyptians, highlighting substantial heterogeneity in both the mechanisms and lived experiences of weight stigma across socio-cultural contexts [42]. Findings from this study, which was carried out in a nationwide sample of adults in Poland, may contribute to the global state of knowledge on social perception of obesity.

Findings from this study revealed significant gaps in public knowledge on obesity and misperceptions of obesity. Gender and age differences in perception of obesity suggest that there is a need to implement educational campaigns that address demographic gaps. Moreover, this study revealed differences in social perception of obesity by BMI status that also justify the need for psychological support for people with obesity. Economic status was also identified as a significant factor affecting social perception of obesity, and public health actions are needed to address beliefs on obesity in low socio-economic groups. Due to the growing burden of obesity in Poland, public health actions are needed to increase public awareness of obesity, its causes and risk factors, as well as the social aspects of obesity.

### Limitations

This study has typical limitations for cross-sectional studies. The study questionnaire was self-prepared based on a literature review, so only selected questions on social perception of obesity were addressed. Items like Fat Phobia Scale (FPS) were not used in this study. The number of questions was limited to the most important ones due to the limited project budget. Data were self-reported, so recall bias may occur. Moreover, the CAWI method was used for data collection, so those without Internet access (around 46% of households in Poland [24]) could not participate in this study. Socio-economic status was not included into stratification model for quota sampling.

In conclusion, among adults in Poland, obesity is perceived as a significant health problem, but misperceptions of obesity are common in society. Almost half of adult Polish residents perceive obesity as a lack of interest in their own body and a reason for shame and have witnessed disrespect towards obese people. Data on social perception of obesity underlines the need to implement public interventions addressing both health and social aspects of this disease.

## Data Availability

Dataset available on request from the authors.
